# View and review on viral oncology research

**DOI:** 10.1186/1750-9378-5-11

**Published:** 2010-05-24

**Authors:** Valeria Bergonzini, Cristiano Salata, Arianna Calistri, Cristina Parolin, Giorgio Palù

**Affiliations:** 1 Department of Histology, Microbiology and Medical Biotechnologies, Division of Microbiology and Virology, University of Padova, Via A Gabelli 63, Padova 35121, Italy; 2 Department of Biology, University of Padova, Via Ugo Bassi 58B, Padova 35123, Italy

## Abstract

To date, almost one and a half million cases of cancer are diagnosed every year in the US and nearly 560,000 Americans are expected to die of cancer in the current year, more than 1,500 people a day (data from the American Cancer Society at http://www.cancer.org/). According to the World Health Organization (WHO), roughly 20% of all cancers worldwide results from chronic infections; in particular, up to 15% of human cancers is characterized by a viral aetiology with higher incidence in Developing Countries. The link between viruses and cancer was one of the pivotal discoveries in cancer research during the past Century. Indeed, the infectious nature of specific tumors has important implications in terms of their prevention, diagnosis, and therapy. In the 21^st ^Century, the research on viral oncology field continues to be vigorous, with new significant and original studies on viral oncogenesis and translational research from basic virology to treatment of cancer. This review will cover different viral oncology aspects, starting from the history of viral oncology and moving to the peculiar features of oncogenic RNA and DNA viruses, with a special focus on human pathogens.

## A brief history of viral oncology

The first evidence of tumor viral aetiology dates back to 1907 when Ciuffo and co-workers showed that human warts could be transmitted by cell-free filtrates derived from lesions [1]. Seventy years later, papillomaviruses were linked to human cancer. In 1908, Ellermann and Bang, reported that also leukemia could be transferred to healthy chicken by a cell-free filtrate of cells obtained form affected birds [2]. Moreover, in 1911, Rous and colleagues showed that the spindle cell sarcoma could be transmitted to healthy chickens using filtered cell-free tumor extracts [3]. This study led to the identification of the first oncogenic virus: the Rous sarcoma virus (RSV). Over the next four decades after the discovery of RSV new tumor viruses were identified. In particular, in 1935, Rous and Beard demonstrated that the cottontail rabbit papillomavirus (CRPV), discovered few years earlier [2], was able to induce skin carcinomas in domestic cottontail rabbit [4]. Moreover, in 1951, studies by Gross and co-workers led to the identification of the first mouse leukemia virus (murine leukemia virus) [5], later confirmed by Moloney and others [6-8], and in 1953 of a mouse virus that induced a variety of solid tumors (mouse polyomavirus) [9]. As far as primates is concerned, in 1962, Eddy, Hilleman, and co-workers showed the tumorigenic potential of simian virus 40 (SV40) [10,11] while, interestingly, Trentin and colleagues reported, for the first time, that viruses could be linked to cancer development also in humans, at least under experimental conditions. Indeed, these authors showed that specific human adenoviruses are tumorigenic in experimentally infected animals [12]. Starting from then, studies were focused on connection among viruses and human cancer. In 1965, Epstein, Barr, and colleagues were able to visualize by electron microscopy herpesvirus-like particles in a cell line, established from Burkitt's lymphoma (BL) [13]. This virus resulted to be biologically and antigenically distinct from other known human herpesviruses [14] and was named Epstein-Barr Virus (EBV). In addition to a causal role in BL, EBV infection has been subsequently associated with nasopharyngeal carcinoma, post-transplant lymphomas, and some Hodgkin's lymphomas (HL), thus representing the first known human tumor virus. In the same year, Blumberg, during a study aimed to correlate inherited polymorphic traits in different geographic areas of the world with particular disease patterns, found that one blood sample from an Australian aborigine contained an antigen that reacted specifically with the serum from an American haemophilia patient. This antigen was named the Australia (Au) antigen and its role was unknown till a technician working with human sera contracted hepatitis, becoming Au antigen-positive. In 1967 and 1968, Blumberg, Okochi, Murakami, and Prince, published seminal studies showing that blood from hepatitis patients contained the Au antigen [15-17], being the surface antigen of a hepadnavirus called hepatitis B virus (HBV), the aetiologic agent of serum hepatitis disease [18]. In 1975, Blumberg and colleagues linked chronic HBV infection to hepatocellular carcinoma (HCC) [19], which is among the most common cancers in the world. Importantly, in 1976 the first effective HBV vaccine was developed by large-scale purification of HBV surface antigen from the serum of chronic HBV carriers [20] followed by a second-generation recombinant HBV surface antigen subunit vaccine produced in 1980 which is still in use. The HBV vaccine protects not only from acute and chronic hepatitis but also from the development of HCC [21,22], thus representing a key achievement in cancer research. The success of the HBV vaccine in decreasing the incidence of liver cancer prompted similar efforts to develop safe vaccines capable of preventing other types of cancers. In particular, the attention was focused on cervical carcinoma. Indeed, in 1974, for the first time, Harald zur Hausen had proposed that the human papillomavirus, HPV, may represent the aetiologic agent for cervical cancer [23,24]. In particular, zur Hausen had demonstrated the presence of novel types of HPV DNA, namely HPV16 and HPV18, in cervical cancers [25,26]. Evidence now clearly indicates that different types of HPV are human tumor viruses responsible for causing virtually all cases of cervical cancer [27], the third leading cause of cancer-related deaths in women worldwide. Furthermore, HPVs have also being linked to other anogenital cancers, as well as to a subset of head and neck cancers. In fact, HPVs are associated with more human cancers than any other virus, causing up to 500,000 cases of cancer per year worldwide [28]. Consequently, HPV has emerged as one of the most important risk factors for human cancer. In 2005 and 2006, large-scale clinical trials with a VLP-based HPV vaccines have been undertaken [28] and current estimates suggest that these HPV vaccines could prevent more than 300,000 cervical cancer cases per year on a global scale.

The most recent milestones in tumor virology have come from the identification of additional human tumor viruses: human T-cell leukemia virus type 1 (HTLV-1), hepatitis C virus (HCV), Merkel cell polyomavirus (MCPyV), and Kaposi's sarcoma virus (KSHV).

In 1980, Gallo and colleagues detected in cultured human T-cell lymphoma cells retroviral reverse transcriptase activity and visualized retroviral particles, immunologically distinct from other known viruses [29]. The new retrovirus was named HTLV-1. In 1981, a causal role for HTLV-1 in adult T-cell leukemia (ATL) was given by the discovery of retroviral particles in cell lines derived from patients [30]. Moreover, patients with ATL but not control individuals were shown to produce antibodies that specifically recognized antigens expressed in HTLV-1-infected human T-cells. Since then different molecular mechanisms behind HTLV-1 oncogenic activity have been undisclosed and will be analysed later on in this review.

In 1989, Houghton and colleagues, by employing an innovative techniques based on phage display library, identified an antigen encoded by a previously unknown RNA virus, which was named HCV [31]. Moreover, using the first serologic test for HCV, Houghton was also capable to confirm that HCV was indeed the aetiologic agent for post-transfusion hepatitis related neither with HBV nor with hepatitis A virus, an other hepatitis virus transmitted by the fecal-oral route, via contaminated food or drinking water [32,33]. In addition, he reported an association between chronic HCV infection with HCC, similar to the one well established for HBV [34,35].

Kaposi's sarcoma (KS) is an endemic tumor of the Mediterranean basin and Africa [36] rarely life-threatening, usually affecting elderly males, with skin localization. In acquired immune deficiency syndrome (AIDS) patients, however, KS displays frequent involvement of extra-cutaneous sites, typically lungs and gastrointestinal tract, with severe complications. Moreover, since AIDS patients exhibited a 20,000-fold higher risk for developing KS, an infectious aetiology for the tumor was suggested. Having epidemiologic and experimental evidence ruled out a role for the human immunodeficiency virus 1 (HIV-1) in this context, the studies were focused on the research of new sexually transmitted infectious agents. As with the search for HCV, a modern molecular biological approach played a central role in the identification of KSHV. Indeed, in 1994, Chang, Moore, and colleagues reported the use of representational difference analysis to identify in 90% of KS tissues from AIDS patients DNA fragments distantly homologous to the herpesvirus EBV [37]. This new virus was called KSHV or human herpesvirus type 8 (HHV-8). Studies carried on in the past 15 years strongly indicate a causal role for KSHV in the development of KS [36], even though the viral infection is clearly not sufficient to produce the disease and other cofactors have been hypothesized.

Finally, a new polyomavirus, MCPyV, has been recently detected in samples derived from patient affected by an aggressive human skin cancer, the Merkel cell carcinoma [38,39].

A summary of the human viruses clearly linked to cancer development is reported in Table 1.

**Table 1:** Human viruses associated with cancer development.

Virus family	Virus	Human tumors	Vaccine
**Papillomaviridae**	HPV16/18	Cervical, Anogenital and Oesophageus tumors	√
**Polyomaviridae**	JCV	Brain and Colon tumors	
	SV40	Mesothelioma and Colon tumors	
	MCPyV	Merkel cell carcinoma	
**Hepadnaviridae**	HBV	Hepatocellular carcinoma	√
**Flaviviridae**	HCV	Hepatocellular carcinoma	
**Herpesviridae**	EBV	Nasopharingeal carcinoma, Burkitt's lymphoma, Hodgkin's lymphoma, B-cell lymphoproliferative diseases	
	HHV-8	Kaposis's sarcoma, Primary effusion lymphoma	
**Retroviridae**	HTLV-1	Adult T-cell leukemia	

## The viral oncogenetic mechanisms: an introduction

Tumor viruses are classified in two general groups based on whether an RNA or DNA genome is packaged into the infectious viral particle. Besides the difference in replication and life cycle, RNA and DNA viruses differ also in their general mechanisms of inducing cellular transformation/immortalization, the first step to tumor development. RNA tumor viruses, in particular animal retroviruses, are usually characterized by the ability to carry and/or alter important cellular growth-regulatory genes, namely the oncogenes. The proteins encoded by these cellular genes are not essential for viral replication and are usually key player in cell cycle control. On the other hand, DNA tumor viruses (like SV40, mouse polyomavirus, adenovirus, and papillomavirus) cause cell transformation by encoding proteins of exclusively viral origin and essential for viral replication.

## Oncogenic RNA viruses: the oncogene discovery

**Figure 1:**
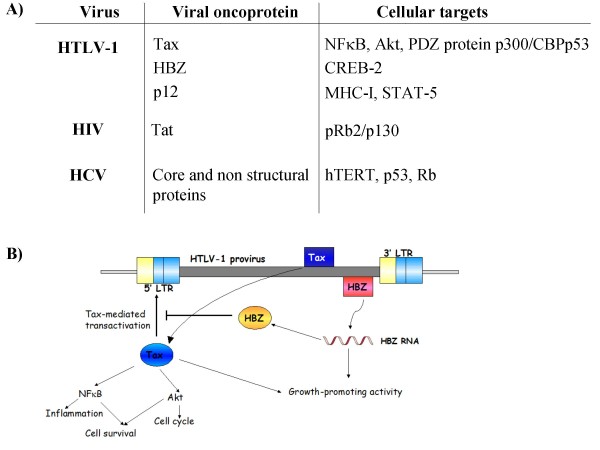
(A) Representative* list of cellular/viral protein interactions involved in RNA virus-related oncogenic transformation; (B) Schematic representation of Tax and HBZ roles in HTLV-1 mediated oncogenesis. Tax modulates the expression of many viral and cellular genes and it also promotes malignant transformation through disruption of different host-cell growth control pathways, resulting in aberrant cell division. Moreover, Tax adversely influences cellular homeostasis through a number of mechanisms, including the physical interaction with cell-cycle regulators and transcriptional activation of cell-cycle control genes, leading to uncontrolled cell division and proliferation. The basic leucine zipper protein (HBZ) is encoded by the complementary strand of the HTLV-1 genome, and it is expressed in all ATL cells, where it is capable of promoting cell proliferation and suppressing Tax-mediated transactivation. LTR: Long Terminal Repeat; NFκB: Nuclear Factor kappa-light-chain-enhancer of activated B cells; MHC-I: Major Histocompatibility Complex Class-I; STAT-5: Signal Transducer and Activator of Transcription-5; hTERT: human Telomerase Reverse Transcriptase. *Additional cellular/viral interactions involved in cell transformation and oncogenetic mechanisms have been described. This list is representative, not exhaustive.

The RNA viruses associated with human cancer are mainly included in Retroviridae and Flaviviridae families (Figure 1).

Animal retroviruses have been characterized earlier, starting from the RSV studies. Indeed, one of the major breakthrough in understanding the molecular mechanisms behind the ability of RNA viruses to cause cancer came from the RSV field. In particular, the observation that cellular transformation and viral replication by RSV were dissociable properties [40] suggested that the virus was encoding a cancer-causing gene, dispensable for viral replication. In 1970, Duesberg and Vogt by comparing the genomes of two closely related replication-competent RSV variants, one of which could transform cells and the other which could not [41] demonstrated that the transformation-competent RSV variant exhibited at the level of the 3'-end additional sequences accounting for a genome 20% larger than the one of transformation-defective RSV variant. This cancer-causing gene (oncogene) was named src according to the type of tumor caused by RSV in chickens, the sarcoma. The established dispensability of the src gene for RSV replication lead to the hypothesis that oncogenes have a cellular origin and that carcinogenic events activate cellular genes to promote cancer. Thus, the reverse transcriptase-dependent life cycle typical of most RNA oncogenic viruses, like RSV, would allow the viral genome to capture a cellular oncogene. In 1976, Bishop and Varmus proved that this hypothesis was correct [42]. Indeed, they were able to obtain src specific probes, starting from transforming RSV genome, and demonstrated its hybridization with the DNA of normal chicken cells and with the DNA of other avian species, even though with lower stringency. This evolutionary conservation of src sequences provided strong evidence that src was indeed a cellular gene acquired by RSV from the chicken genome, rather than being a viral gene. Moreover, this finding suggested that the cellular gene, designated a proto-oncogene, must sustain a mutation to cause cancer thus associating tumors with mutagenic events. Supporting this view, it was then demonstrated that ras oncogenes present in human bladder carcinoma cell lines, and rat mammary carcinomas contained a mutation crucial for inducing cellular transformation absent in ras proto-oncogene present in normal cells [43,44]. Viral oncogenes carry as well mutations or are constitutively expressed with negative effects on cell proliferation control. Indeed, to date, more than 70 cellular proto-oncogenes have been identified through studies of oncogenic retroviruses, and nearly all of these genes code for key cell signaling proteins involved in the control of cellular proliferation and apoptosis [45]. The ability to encode viral oncogenes is not the only mechanism by which RNA viruses can cause cancer. It has been demonstrated that retroviruses not carrying oncogenes in their genome may influence expression/function of cellular oncogenes by insertional mutagenesis [45].

Among human retroviruses, the earliest oncogenic virus identified was HTLV-1, associated with the ATL, an aggressive clonal malignancy of mature CD4+ T lymphocytes that presents after a 20-40 years period of clinical latency [46]. Interestingly, HTLV-1 still represents the only known human retrovirus directly linked to a specific human malignancy. Indeed, several epidemiological and molecular evidence implicates HTLV-1 as the aetiologic agent of ATL: i) the geographic distribution of ATL in different world areas, from Japan to Central African, the Caribbean basin, Taiwan, and Papua New Guinea matches that of HTLV-1 infections [47]; ii) when checked, ATL patients always underwent HTLV-1 infection; iii) leukemic cells cultured derived from ATL patients are positive for HTLV-1; iv) HTLV-1 infection of normal human T cells induced cellular transformation and immortalization.

Interestingly, by contrast to mechanisms typical of animal retroviruses, HTLV-1 does not cause cancer by insertional mutagenesis or by capturing and activating cellular proto-oncogenes. Rather, the major oncogenic determinant of HTLV-1 is the viral Tax gene that encodes a protein essential for viral replication [48]. In particular, it has been demonstrated that Tax associates with centrosomes, causing their fragmentation [49], and it is crucial for transactivation/repression of viral and cellular gene expression. Oncogenic transformation of infected and transfected cells would hence be due to the interaction with various transcription factors [50]. Furthermore, Tax induces genome instability by deregulation of cell cycle checkpoints. According to the chromosomal alterations observed in ATL patients, it has been described that Tax is able of inducing a delay in the cellular recognition and response to DNA damage, and of suppressing the DNA repair machinery activation [51]. Thus, Tax drives genome instability and cellular transformation by interfering with cell cycle checkpoint pathways and DNA repair mechanisms. Hence, Tax can be considered a viral oncoprotein, since Tax alone transforms rat fibroblasts and primary human T lymphocytes, while transgenic mice expressing Tax develop tumors [52]. Moreover, recent evidence supports a role for the HTLV-1 basic leucine-zipper factor (HBZ) as an additional viral player in cancer development [53,54]. Interestingly, HBZ expression in transgenic mice confers a phenotype similar to the one observed in ATL patients, and in particular the infiltration of lymphocytes into skin and lung [53,54]. A schematic representation of HBZ role in HTLV-1 mediated oncogenesis is reported in Figure 1. Finally, it has been shown that HTLV-1 is able of altering the major histocompatibility complex class-I (MHC-I) and the T cell receptor (TCR) cascade activation through the accessory protein p12. p12 targets the free MHC-I chain and increases Signal Transducer and Activator of Transcription-5 (STAT-5) protein activation and calcium release [55]. Furthermore, p12 decreases viral expression in TCR-stimulated T cells and is recruited to the immunological synapse [56]. Besides, HTLV-1 accessory proteins p13II is able to target the mitochondria and to induce changes in its morphology. In particular, the effects on mitochondria result mainly at the membrane permeability level, altering the inner membrane potential, and the oxygen consumption (respiration), thus affecting cellular proliferation, apoptosis, and reactive oxygen species (ROS) production [57,58].

The hypothesis of an HIV-1 involvement in tumor pathogenesis is based on the evidence that AIDS-related tumors have been described, such as KS, non-Hodgkin's lymphomas (NHLs), and invasive cervical carcinoma (ICC) [59]. HIV-1 infection can play a direct and/or indirect role in HIV-1-related tumorigenesis. Among the HIV-1 proteins of particular importance with regard to a possible role in the carcinogenesis is the accessory protein Tat [60]. In this context, it has been proposed that the Tat-induced DNA repair deficiencies may play a significant role in the development of AIDS-associated cancers [60]. In particular, in the case of ICC, HIV-1 Tat, besides enhancing the activity of HPV oncogenes, by upregulating HPV E6 and E7 gene expression [61], could also promote cell cycle progression [61]. Moreover, it has been suggested that Tat, by physically interacting with pRb2/p130, might alter pRb2/p130 cell growth-suppressive properties, leading to the loss of cell cycle control [62].

Besides, considering clinical data about HIV-1-associated primary cerebral lymphoma, several important differences of AIDS to non-AIDS related primary cerebral lymphomas have been described [59,63,64]. Among them are the higher aggressiveness, the presence of multi-focal lesions, the reduced percentage of therapy responders, and an elevated mortality [63,64].

In addition to the retroviruses, HCV, a member of the Flaviviridae family, is associated with human cancer. HCV infection affects more than 170 million individuals worldwide and represents one of the main causes of chronic liver disease (CLD) that can evolve in HCC [65]. Among patients infected with HCV, it is almost exclusively those with cirrhosis (roughly 20%) who develop HCC, revealing a major risk factor for malignant progression. For these patients, the annual risk for developing HCC is 1% to 4%, with patients from Japan having an even higher risk. Chronic inflammation and cirrhosis are believed to play key roles in promoting HCV-associated HCC, although the underlying mechanisms of this process are not yet understood. In addition to HCC, HCV is also involved in polyclonal B lymphocyte activation [66] and epidemiological studies show that HCV seropositive individuals have a 5.5 times higher risk developing NHL compared to HCV-seronegative individuals [67]. Clonal B cells may evolve to overt HCV-related NHL as result of an antigen-driven process triggered by the E2 protein [66,67]. Noteworthy, the characterization of clonal B cells activation mechanisms may represent a suitable target to develop a therapy for HCV-associated NHL [66,67].

## Oncogenic DNA viruses: the discovery of tumor suppressors

By contrast to RNA viruses, usually oncogenes of DNA tumor viruses lack any recognizable sequence similarities to cellular genes and how the products of these viral genes were able to transform cells was not elucidated till late 1970s [68,69]. It has been demonstrated few years earlier that SV40 was capable to induce tumor formation in experimentally infected hamsters, by the expression of the viral large tumor (T) antigen, the major oncogenic determinant of SV40 [70-72]. By employing co-immunoprecipitation techniques, it was shown that the SV40 large T antigen was interacting with a cellular protein having an approximate molecular weight of 53 kDa. Based on its size, this cellular protein was named p53. This finding represented the first evidence that products of DNA tumor virus oncogenes could function through physical/direct interactions with cellular proteins. By cloning p53 genes from neoplastic rodent and human cells it was possible to demonstrate that in all cases the coding sequences differed from those present in normal cells, by carrying important gain-of-function mutations. Indeed, p53 is mutated or lost in almost 50% of all human cancer cases worldwide, representing the most commonly mutated gene in human tumors. This finding suggests that p53 acts as a tumor suppressor gene, which in contrast to proto-oncogenes function to prevent rather than to promote cancer [73]. Several studies have contributed to demonstrate that a wide variety of cellular stress stimuli, such as DNA damage but also viral infection, induce the activation of p53, which binds to and regulates the activity of several important cellular factors [73]. In this way, p53 controls cell cycle progression, senescence, apoptosis, and DNA repair thus preventing tumor formation by reducing the accumulation of genetic lesions. In the case of viral infection p53 activation represents the attempt of the host cell to block viral replication, by inducing, for instance, apoptosis. Thus, several DNA viruses have evolved proteins, such as the SV40 large T antigen, to bind and inactivate p53, in order to escape the cellular antiviral response [45], with cell transformation as a consequence.

Inactivation of p53 is not the only mechanisms evolved by DNA oncogenic viruses which induces tumors. A second tumor suppressor genes, was discovered by studying the childhood tumor retinoblastoma (Rb) [74]. Rb susceptibility was linked to a single recessive trait and the gene encoding the specific tumor suppressor gene was identified and cloned [75,76] and the protein named Rb. In 1988, Harlow, Livingston and co-workers, demonstrated that the Rb protein immunoprecipitates with adenovirus E1A and with SV40 large T antigen from transformed cells [77,78].

Studying the interactions among human adenovirus E1A and SV40 large T antigen with Rb was essential for understanding the cellular tumor suppressor function [79] with the demonstration that a hypophosphorylated form of Rb negatively regulates G1 to S phase progression through the cell cycle by binding to and blocking the activity of E2F, a transcriptional factor activating several genes involved in cellular DNA replication. Cell progression through G1 to S phase depends on the G1 cyclin-dependent kinases activity, which directly hyperphosphorylate and inactivate Rb leading to the release of active E2. Viral oncoproteins specifically bind to and inactivate the hypophosphorylated form of Rb. Thus free active E2F accumulates, with consequent uncontrolled cellular proliferation.

**Figure 2:**
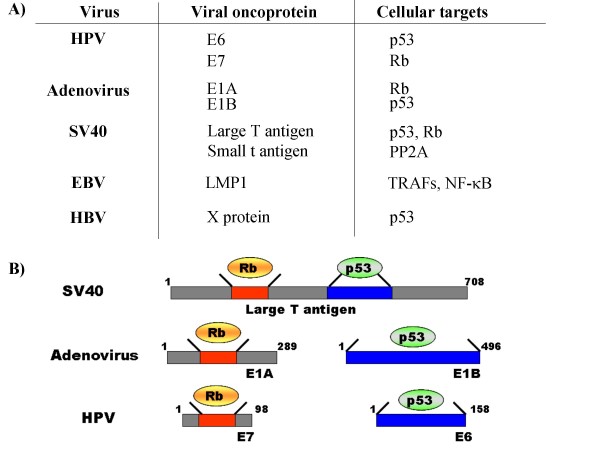
(A) Representative* list of cellular/viral protein interactions involved in DNA virus-related oncogenic transformation; (B) p53 and Rb are central targets of viral oncoproteins. The viral proteins Large T antigen of SV40, E1A/E1B of Adenovirus, and E6/E7 of HPV, are capable of interfering with either Rb and/or p53, altering their activities and thus essential cell cycle check-points. PP2A: Protein Phosphatase 2A; TRAFs:Tumor necrosis Factor Associated. * Additional cellular/viral interactions involved in cell transformation and oncogenetic mechanisms have been described.

In summary, studies of DNA tumor viruses provided seminal contributions to our understanding of both Rb and p53, two of the most important cellular tumor suppressor proteins. Moreover, a common theme for DNA tumor viruses emerged since it has been demonstrated that oncoproteins encoded by SV40, adenovirus, and HPV share similar capacities for inactivating both the Rb and p53 tumor suppressors [45]. A schematic representation of DNA viruses and related cellular/viral genes involved in oncogenesis is reported in Figure 2.

To date, the DNA viruses consistently associated with human tumors, are the HBV, HPV, EBV, and HHV-8 [80,81]. In addition, several evidence suggest a causative role in some human cancers also for SV40, BK Virus (BKV) and JC Virus (JCV) [80-83].

HPV represents a typical example of human oncogenic DNA virus. Specific genotypes have been clearly linked to different forms of tumors, mainly cervical cancer, but also some penile and upper aerodigestive tract carcinomas [45,84]. Being the viral aetiology of the above tumors so well established, as for HBV, it is expected that the recently tested HPV vaccines [45,84] will have a profound impact on their prevention [45,84-90]. The HPV E6 and E7 oncoproteins play an important role in cervical tumors development, and are continuously expressed in the lesions, while tumor arises only several years after the initial cellular immortalising events. In fact, the continuous expression of E6 and E7 is required for maintenance of the transformed phenotype, and prevention of cell growth arrest and/or apoptosis [91,92]. The best-characterised HPV16 E6 activity is its ability to induce degradation of the tumour suppressor protein p53 via the ubiquitin pathway [93]. Moreover, among additional functions, E6 protein can also interfere with cellular differentiation and cell cycle progression [94]. E7 is an acidic phosphoprotein of 98 amino acids, which is structurally and functionally related to a gene product of other DNA tumour viruses, the adenovirus E1A protein and SV40 large T antigen. As mentioned above, all three proteins are capable of binding to the tumour suppressor protein retinoblastoma (pRb1) and its related proteins p107 and p130, involved in cell cycle regulation. By doing so, and thanks to other functions, E7 controls cell cycle progression [94]. In addition to E6 and E7, HPV E5 protein, a small hydrophobic protein, localized in the endomembrane compartments of the Golgi apparatus and endoplasmic reticulum could play a role in HPV carcinogenesis. Indeed it has been demonstrated that HPV E5 down-regulates surface MHC-I, thus preventing its transport to the cell surface; hence, E5 can potentially allow infected cells to escape adaptive immune response of cytotoxic T lymphocytes (CTL), thus favouring viral persistence [95,96].

As for HCV, HBV chronic infection represents an important risk factor for the development of HCC, a malignant tumor frequently observed in some countries of Asia and Africa [97,98]. It is important to underline how major achievements in the prevention of virus-induced cancers may be attributable to strategies to control infection in human populations. In fact, HBV vaccination has dramatically decreased the number of HCC [45,99-101]. However, the molecular mechanisms of HBV carcinogenesis are still not fully clarified. Different studies suggest a role for the hepatitis B virus × antigen (HB×Ag) in this context. Indeed, in addition to a physical binding and functional inactivation of p53, HB×Ag promotes fibrogenesis by stimulating fibronectin expression, inhibits apoptosis mediated by Fas and tumor necrosis factor-alpha (TNF-α) and its expression correlates with the development and progression of CLD [102,103]. In particular, HB×Ag expressed by HBV genome integrated into chromosomal DNA is often functional in trans-activation assays [102]. Moreover, it can alter patterns of host gene expression, contributing to carcinogenesis, by activating signal transduction pathways in host-infected cells [102,103]. HB×Ag transforms cell lines *in vitro*, giving rise to liver cancer in transgenic mice [103]. A role in the ability of HB×Ag to modulate specific cell pathways has been linked to the upregulated gene clone 11 (URG11) cellular protein, which appears to be a direct effector of the viral protein. Indeed, URG11 is upregulated in HB×Ag-positive liver cells and seems to be involved in cancer evolution, by controlling cell cycle progression [103].

There is emerging interest in the polyomaviruses as possible human carcinogens [80,83,104-107]. SV40, which naturally infects the rhesus monkey, was inadvertently introduced into the human population as a contaminant of early poliovirus vaccines, whereas the BK and JC polyomaviruses are natural human pathogens associated with disease processes in the urinary tract or brain, respectively. Genomic sequences of these three polyomaviruses, which are tumorigenic under experimental conditions, have been detected in human mesothelioma, osteosarcoma, NHL, brain tumors, and prostate cancer. In addition, an integrated form of a new polyomavirus, MCPyV, was recently observed in Merkel cell carcinoma, a rare but aggressive human skin cancer of neuroendocrine origin [39,84].

EBV and HHV-8 are two members of the Herpesviridae family that are classified as cancerogenic agents. These viruses can establish long-term viral infections in their target cells, promoting cellular immortalisation and transformation [80,81]. EBV is the most important aetiological factor in classic BL [108], and it is also detectable in undifferentiated nasopharyngeal carcinomas, in a subset of HL, and in some cases of NHL, notably in immunosuppressed patients [79,80,108].

The alteration of cell signaling represents the molecular basis for cellular proliferation occurring in association with several viral infections. In particular, both EBV and HHV-8 target important cell signaling pathways involved in oncogenesis, such as the β-catenin pathway that plays a key role in the control of cell adhesion and tissue morphogenesis [109,110]. The level of β-catenin protein is subject to tight regulation, particularly through ubiquitin-mediated proteasomal degradation. Latent membrane protein-1 (LMP-1) and latent membrane protein-2A (LMP-2A) of EBV affect the β-catenin stabilization and activation avoiding the ubiquitination [110], as many other oncoproteins of tumorigenic viruses, such as T antigen in JCV [105]. It is beginning to emerge that tumor viruses modulate the ubiquitination of specific cell factors for their needs [111,112] by employing different strategies. Among them, viruses encode their own ubiquitin ligases and deubiquitinating enzymes (DUBs), as recently demonstrated in the case of herpesviruses [112,113]. Indeed, since the discovery that the largest tegument protein of Human Herpes Virus-1 (HSV-1), UL36, contains a deubiquitinating activity it has been reported that all members of the Herpesviridae family, including EBV and HHV-8, encode UL36 homologues, suggesting an important role of this protein in the viral life cycle [111,114]. On the other hand, the presence of the viral latency-associated nuclear antigen (LANA) in all HHV-8-associated tumors significantly correlates with β-catenin over-expression. In this context it has been demonstrated that introduction of anti-LANA small interfering RNA (siRNA) into primary effusion lymphomas (PEL) cells eliminated β-catenin accumulation, while LANA itself upregulated expression of β-catenin in transfected cells. LANA stabilizes β-catenin by binding to its negative regulator GSK-3β, causing a cell cycle-dependent nuclear accumulation of GSK-3β [115]. The importance of this pathway to HHV-8-driven cell proliferation is highlighted by the observation that LANA stimulates entry into S phase.

In the past 10 years much effort has been devoted to the study of HHV-8. HHV-8 is consistently detected in all forms of KS, in PEL, and in a subset of multicentric Castleman's disease [116,117]. During the latency program of γ-herpesvirus infection, few viral genes are expressed. Whereas EBV latent proteins contribute to cell immortalization, HHV-8 lytic genes play an important role in cancer development and progression. An Italian study investigated the latent and lytic antibodies seroprevalence in elderly subjects, and the possible correlation with clinical stage and disease progression in classical KS [118,119]. While the antibody levels against HHV-8 latent antigens were observed in all KS cases, antibody levels against HHV-8 lytic antigens increase with the progression of KS, and higher HHV-8 antibody levels were observed in the fast progressive form of the disease [119]. According to the literature, these results support the hypothesis that active viral replication probably contributes to progression of KS. In addition, HHV-8 DNA was constantly detected in saliva and PBMC samples of classical KS but without any correlation with the clinical stage of the disease, suggesting that oral shedding is likely to occur in these patients and contributes to viral transmission [119].

## Conclusive remarks

By the first decade of the 21st Century, much evidence had accumulated pointing out at least six human viruses, namely HPV, HBV, EBV, HHV-8, HCV, and HTLV-1, as aetiologic agents of human cancers. As mentioned above these viruses are responsible of roughly 20% of all human tumors worldwide [45]. Moreover, oncogenic viruses have also proved to be powerful tools for dissecting fundamental pathways and proteins involved in cell cycle progression and regulation. For example, a number of oncogenes have been identified through studies focused on RNA tumor viruses, while essential tumor suppressors, such as p53 and Rb, were discovered and characterized through DNA tumor viruses. In the future, it is expected that the characterization of new tumor viruses will contribute in clarifying relevant aspects of cell biology and carcinogenesis. Indeed, other candidate human tumor viruses have been proposed [120]. Particularly intriguing in this context is the study of human endogenous retroviruses role in seminomas, breast cancer, myeloproliferative disease, ovarian cancer, melanoma, and prostate cancer [121]. Moreover, as demonstrated in the case of HBV and HPV, prophylactic vaccines offer the potential to prevent cancers having a viral aetiology. Thus, the development of new vaccines against other human tumor viruses should be a must for the future research.

## Competing interests

The authors declare that they have no competing interests.

## Authors' contributions

All authors have contributed in writing the review. All the authors read and approved the final manuscript.
